# A Bivariate Hypothesis Testing Approach for Mapping the Trait-Influential Gene

**DOI:** 10.1038/s41598-017-10177-5

**Published:** 2017-10-09

**Authors:** Garrett Saunders, Guifang Fu, John R. Stevens

**Affiliations:** 1 Department of Mathematics, Brigham Young University-Idaho, Rexburg, ID 83460 USA; 2 Department of Mathematics and Statistics, Utah State University, Logan, UT 84322 USA

## Abstract

The linkage disequilibrium (LD) based quantitative trait loci (QTL) model involves two indispensable hypothesis tests: the test of whether or not a QTL exists, and the test of the LD strength between the QTaL and the observed marker. The advantage of this two-test framework is to test whether there is an influential QTL around the observed marker instead of just having a QTL by random chance. There exist unsolved, open statistical questions about the inaccurate asymptotic distributions of the test statistics. We propose a bivariate null kernel (BNK) hypothesis testing method, which characterizes the joint distribution of the two test statistics in two-dimensional space. The power of this BNK approach is verified by three different simulation designs and one whole genome dataset. It solves a few challenging open statistical questions, closely separates the confounding between ‘linkage’ and ‘QTL effect’, makes a fine genome division, provides a comprehensive understanding of the entire genome, overcomes limitations of traditional QTL approaches, and connects traditional QTL mapping with the newest genotyping technologies. The proposed approach contributes to both the genetics literature and the statistics literature, and has a potential to be extended to broader fields where a bivariate test is needed.

## Introduction

Quantitative trait loci (QTL) mapping, an approach to identify and map genetic loci that have significant effects on a quantitative phenotype, has received considerable attention for many years because it can provide close insights about how genetic variants act on phenotypic variation, increase accuracy of estimating genomic position, improve understanding of biological processes, and help prepare for the eventual cloning of the locus^1–3^. With the rapid development of high-density genotyping technology, the demands on QTL research come to a new level^3^.

There are three possible genotypes (*AA*,*Aa*, and *aa*) for a bi-allelic QTL with alleles *A* and *a*. To detect the existence of a QTL, a common strategy tests to see if the phenotypic means among the three genotypes are significantly different from one another. Based on the possible linkage of the QTL with an observed genetic marker, three scenarios (‘no QTL’, ‘unlinked QTL’, and ‘linked QTL’) were defined by Churchill *et al*.^1,4^. Although the literature widely found these three scenarios to have dramatic differences in genetic interpretations and likelihood structures, traditional QTL approaches have failed to truly separate the differences of ‘linkage’ and ‘QTL effect’.

Traditional QTL models perform only one hypothesis test related to the existence of a QTL, while using a recombination fraction *r* to model but not simultaneously test the linkage between the observed marker and the QTL^1,3–5^. The one-test models have a few limitations, however, which are summarized in the following points: 1) They fail to distinguish between a case of ‘large QTL effect but loose linkage with the marker’ and a case of ‘small QTL effect but tight linkage with the marker’. Only estimating the recombination parameter *r* without testing it does not allow inspecting the significance of linkage strength between the QTL and the marker being tested^1,5^. 2) They require that genetic maps are known (a fundamental prerequisite to estimate the recombination fraction *r*), which is generally only possible for classic cross design like F2 intercrosses or backcrosses or well-studied domesticated species. Natural populations, complex designs, and rarely-studied new species require new QTL models^6^. 3) They can not accurately conclude if there is an influential QTL around the observed marker or if an influential QTL is called just by random chance. 4) They were designed for a small to moderate number of genetic markers. Contemporary genomic research, however, demands modern QTL methodologies that are capable of analyzing the entire genome dataset with more than half a million markers.

Besides the general QTL existence test, another linkage disequilibrium (LD) strength test can be used to examine if the QTL and the observed genetic marker are linked^7–12^. The LD based QTL mapping model involves two indispensable hypothesis tests, having the same aim as the traditional *r*-based QTL model but with fewer restrictions and more capabilities. Being consistent with the literature that unanimously consider ‘linked QTL’ as the alternative hypothesis^1,4^, the QTL existence null and the LD strength null must be jointly rejected to conclude the same alternative statement. The two-test structure tests not only how strong a genetic effect is but also where the gene is located; and whether there is a QTL around the observed marker instead of just having a QTL by random chance. However, in some preliminary work the type I error rate of this two-test structure was found to be unexpectedly high due to some open statistical issues involving inaccurate asymptotic distribution s of the test statistics involved.

In this article, we propose a bivariate null kernel (BNK) hypothesis testing method to characterize the joint distribution of the two test statistics in two dimensional space. The novel contributions of the proposed BNK approach are: (1) proposes an important bivariate hypothesis testing approach in two dimensional space, which is not only new to the genetics literature but also important to the statistics hypothesis testing field, and has the potential to be extended to a general setting where a bivariate test is needed. (2) closely divides the genome into several categories by closely separating the confounding between ‘linkage’ and ‘QTL effect’, and provide a clearer picture for the entire genome. (3) solves a few open statistical questions that were noticed and discussed but never solved before, without requiring the distribution of the test statistics to be known. (4) overcomes the limitation of traditional QTL approaches and serves as a bridge connecting QTL research with the newest genotyping technologies. The proposed BNK approach is verified to perform well in three dramatically different simulation designs. The BNK approach performs nicely not only in the LD-based QTL model from which it was developed, but also in a simulation that was designed specifically for traditional recombination based one-test QTL methods. Additionally, the BNK approach performs well not only in genetic data, which is how this approach is motivated, but also in general bivariate normal data with broader applications. We also demonstrate in a real mouse study that the BNK approach selects two genes previously reported to be highly associated with the *HDL* trait being analyzed. In addition, the BNK approach detects six other genes that were found important for homeostasis/metabolism expression, but that had not previously been detected from the same dataset.

## Key Statistical Issues

### Two-test structure in LD-based QTL model

In light of possible relations of a QTL with the observed marker, the corresponding likelihoods for three possible scenarios (‘linked QTL’, ‘unlinked QTL’, and ‘no QTL’) can be most appropriately summarized in the following (same three scenarios as Churchill *et al*.^1,4^):

- *H*_*A*_: linked QTL
  1 $$L(p,q,D,{\mu }_{1},{\mu }_{2},{\mu }_{3},\sigma |Y,M)=\prod _{i=1}^{n}\sum _{g=1}^{3}{\omega }_{g|{M}_{i}}(p,q,D)f({Y}_{i}|{\mu }_{g},\sigma \mathrm{).}$$
- $${H}_{0}^{2}$$: unlinked QTL
  2 $$L(q,{\mu }_{1},{\mu }_{2},{\mu }_{3},\sigma |Y)=\prod _{i=1}^{n}\sum _{g=1}^{3}{\omega }_{g}(q)f({Y}_{i}|{\mu }_{g},\sigma \mathrm{).}$$
- $${H}_{0}^{1}:$$ no QTL

3 $$L(\mu ,\sigma |Y)=\prod _{i=1}^{n}f({Y}_{i}|\mu ,\sigma \mathrm{).}$$

Here, the phenotypic measurement for individual *i*, *Y*_*i*_, $i=\mathrm{1,}\ldots ,n$, is a random variable resulting from genotype *g* of the hidden QTL (having allele *A* or *a* with frequency *q* or 1-*q*), where $g\in \{\mathrm{1,2,3}\}$, with *g* = 1 representing *aa*, *g* = 2 representing *Aa*, and *g* = 3 representing *AA*. $f({Y}_{i}|{\mu }_{g},\sigma )$ denotes the corresponding density functions for the distinct QTL genotypes with mean *μ*_*g*_ and variance ${\sigma }^{2}$, which is generally assumed to be normal for a quantitative trait. Let *M* (with allele frequency *p*) and *m* denote the two alleles of an observed marker. Together, the marker and the QTL form four haplotypes (*MA, Ma, mA*, and *ma*) with corresponding frequencies ${p}_{11}=pq+D$, ${p}_{10}=p\mathrm{(1}-q)-D$, ${p}_{01}=\mathrm{(1}-p)q-D$, and ${p}_{00}=\mathrm{(1}-p\mathrm{)(1}-q)+D$, respectively^12^. Here, *D* is the linkage disequilibrium parameter between the marker and the QTL, which can be estimated as $D={p}_{11}-pq={p}_{11}{p}_{00}-{p}_{01}{p}_{10}$. The mixing proportions ${\omega }_{g|{M}_{i}}$ in likelihood (1) denote the conditional probabilities of individual *i* having QTL genotype *g* given their marker genotype *M*_*i*_, which are functions of *p*, *q*, and *D* and were given in Table 1 of Saunders *et al*.^12^. The mixing proportions ${\omega }_{g}(q)$ in likelihood (2) can be computed as ${\omega }_{1}(q)={\mathrm{(1}-q)}^{2}$, ${\omega }_{2}(q)=q\mathrm{(1}-q)$, and ${\omega }_{3}(q)={q}^{2}$ because the marker and QTL are not linked. Note that likelihood (1) differs from likelihood (2) as the mixture proportions ${\omega }_{g|{M}_{i}}$ are conditional upon the marker information of each individual, thus making the mixture subject-specific.

**Table 1:** Type I error rate and power for the BNK approach obtained under the LD-based QTL mapping framework (simulation 1).

	*n* = 100	*n* = 300	*n* = 500
Power	0.36	0.79	0.98
Type I Error Rate	0.03	0.03	0.03

The first test, concerning the existence of a QTL, is proposed as^1,4,5,11,12^

$${H}_{0}^{L}:{\mu }_{1}={\mu }_{2}={\mu }_{3}\equiv \mu \quad {\rm{vs}}$$

${H}_{1}^{L}$: one of the equalities above does not hold.

It can be seen that under ${H}_{0}^{L}$ the likelihood is given by that of ${H}_{0}^{1}$, i.e., l ikelihood (3). This follows from the fact that under ${H}_{0}^{L}$, $f({Y}_{i}|{\mu }_{g},\sigma )=f({Y}_{i}|\mu ,\sigma )$ for *g* = 1, 2, 3 which then eliminates the parameters *p*, *q*, and *D* from the full model, as ${\sum }_{g=1}^{3}{\omega }_{g|{M}_{i}}(p,q,D)=1$. The alternative ${H}_{1}^{L}$, which suggests the heterogeneity model, corresponds to *H*_*A*_ with l ikelihood (1). Thus, in the first test, as is traditionally done, ${H}_{0}^{1}$ is tested against *H*_*A*_, where the likelihood ratio test is used as the test statistic^1,3–5^:

4 $${T}_{L}=\,\mathrm{ln}[\frac{L(p,q,D,{\mu }_{1},{\mu }_{2},{\mu }_{3},\sigma |Y,M)}{L(\mu ,\sigma |Y)}]\mathrm{.}$$

Under traditional likelihood theory^13^, *T*_*L*_ would be distributed asymptotically as a ${\chi }_{5}^{2}$ random variable because the difference in the number of free parameters between the null ($\mu ,\sigma$) and alternative models (${\mu }_{1},{\mu }_{2},{\mu }_{3},\sigma ,p,q,D$) is five.

The second test, concerning the LD strength of the QTL and the observable marker^7–12^, is proposed as

$${H}_{0}^{D}:D=0\quad {\rm{vs}}\quad {H}_{1}^{D}:D\ne 0.$$

The case of *D* = 0 corresponds to no linkage between the marker and QTL, in which case the conditional probability ${\omega }_{g|{M}_{i}}$ reduces to ${\omega }_{g}$, eliminating marker and subject-related subscripts. Then the test ${H}_{0}^{D}$ allows to step back to ${H}_{0}^{2}$ (with l ikelihood (2)) before officially concluding *H*_*A*_. The alternative ${H}_{1}^{D}$, which suggests the ‘linked QTL’ model, corresponds to *H*_*A*_ with l ikelihood (1). Churchill and Doerge stated more details about this null hypothesis ${H}_{0}^{2}$ for the recombination-based QTL model under a back-cross design^4^, “Under the null hypothesis ${H}_{0}^{2}$, the trait should follow a normal mixture distribution with mixing proportions equal to 1/2. Again any associations between the trait values and markers unlinked to the QTL are due to chance.” The LD-based QTL model has very similar interpretations and contexts for ${H}_{0}^{2}$ as in the more traditional recombination-based QTL model, with two key differences: 1) a clear hypothesis test about linkage strength (i.e., ${H}_{0}^{D}$) is performed rather than only examining (but not testing) the recombination rate *r*; 2) the mixing proportions ${\omega }_{g}$ are a function of *q* (the allele frequency of the QTL) rather than being treated as fixed constant s. These two differences enable the LD-based QTL model to have advantages over recombination - based QTL models, which are described previously in the Introduction section.

While originally developed for evaluating the linkage between two observed markers^14–19^, the test statistic

5 $${T}_{D}=\frac{n{\hat{D}}^{2}}{\hat{p}\mathrm{(1}-\hat{p})\hat{q}\mathrm{(1}-\hat{q})}$$

can also evaluate the linkage between the unobserved QTL and the observed marker. Here $\hat{p}$, $\hat{q}$, and $\hat{D}$ are the estimated allele frequencies of marker, QTL, and the LD between them, respectively. *T*_*D*_ is equivalent to the log likelihood ratio test statistic of ${H}_{0}^{2}$ against *H*_*A*_. *T*_*D*_ estimated from two observable markers was previously claimed to asymptotically follow the ${\chi }^{2}$ distribution with one degree of freedom^14–16,19^.

The bivariate testing structure of the LD-based QTL model is (${H}_{0}^{L},{H}_{0}^{D}$). To be consistent with the biological interpretations and unanimous aim of QTL research, a significant QTL associated with the phenotype is not detected unless the bivariate test $({H}_{0}^{L},{H}_{0}^{D})$ jointly rejects, i.e., the ‘linked QTL’ is detected.

#### Unidentifiability issue of a parameter

The parameter *D* contained in the alternative model (1) for ‘linked QTL’ is not defined in the null model. This causes an ‘unidentifiability’ issue, as mentioned by Davies^20,21^. As a result, the distribution of test statistic *T*_*D*_ will not be clear.

#### Inaccurate asymptotic distributions of the two marginal test statistics

*For the first QTL existence test (* ${H}_{0}^{L}$ *):* Lander and Botstein assumed an asymptotic ${\chi }^{2}$ distribution for its test statistic *T*_*L*_^5^. Their use of the asymptotic ${\chi }^{2}$ distribution has been criticized by Churchill and Doerge, who stated the following: “In most cases, the regularity conditions that ensure an asymptotic ${\chi }^{2}$ distribution for the likelihood ratio test statistic are not satisfied”^3,4^. A simulation study by Knott and Haley generated an F2 intercross, based on the recombination -based QTL model, to assess the goodness of fit of the empirical distribution to the corresponding theoretical asymptotic distribution of the log likelihood ratio test statistic *T*_*L*_^1^. Their results suggest that the ${\chi }^{2}$ approximation to the distribution of likelihood ratio test statistics is not reliable in many cases^4^. Additionally, the challenges of the likelihood ratio test for the normal mixture models have also been confirmed in other settings in the statistics literature^22,23^. To overcome such inaccurate assumptions about the distribution of test statistics *T*_*L*_:, Churchill and Doerge (1994) proposed using permutations to obtain a critical threshold, rather than employing an asymptotic ${\chi }^{2}$ distribution.

*For the second linkage strength test (* ${H}_{0}^{D}$ *):* Although the ${\chi }_{1}^{2}$ distribution of the test statistic *T*_*D*_ worked well, it was claimed for two observable markers^11,14–16,19^. As a matter of fact, the linkage of one hidden QTL and one observable marker is a different scenario. The simulation study of Knott and Haley found overwhelming evidence that the log likelihood ratio test statistic of ${H}_{0}^{2}$ against *H*_*A*_, using the *r*-based QTL model, was not distributed as a ${\chi }_{1}^{2}$ ^1^. These results were also confirmed later on by Luo *et al*. using the LD-based QTL model^7^. As suggested in Luo *et al*.^7^, who studied the likelihood ratio test statistic (an equivalent statistic to that of *T*_*D*_), it is not clear what the distribution of the test statistic in the context of an observed marker and a hidden QTL is, but it is not a ${\chi }_{1}^{2}$. The distribution of test statistic *T*_*D*_ for ${H}_{0}^{D}$ with one hidden QTL and another observable marker is still an open statistical question. Consequently, the type I error rate is inflated, which was observed, but no one has yet solved the issue.

#### Confirming the inaccurate distributions of two marginal test statistics via simulation

As discussed above, the asymptotic distribution of univariate test statistics, either *T*_*L*_ or *T*_*D*_, has been found problematic in various models. In this sub section, we empirically confirm the same inaccuracy for the two marginal test statistics, *T*_*L*_ and *T*_*D*_, via simulation on the two-test structure in the LD based QTL model, which is the motivating setting of this article.

As a simulation example, we reimplemented the *F*_2_ intercross simulation study of Knott and Haley^1^. A phenotype $Y \sim N\mathrm{(0,1)}$ and single marker *M* with allele frequency of *p* = 0.5 were generated independently, 1,000 times under a sample size of *n* = 500 (i.e., generated data under ${H}_{0}^{1}$). For each replication pair of *Y* and *M*, the test of ${H}_{0}^{L}$ was performed to compute *T*_*L*_ and the test of ${H}_{0}^{D}$ was also performed to compute *T*_*D*_. Additionally, a separate simulation was conducted where the marker and an observed QTL generated by random chance were simulated independently (i.e., generated data under ${H}_{0}^{2}$), and only a test of ${H}_{0}^{D}$ was performed to explore the distribution of *T*_*D*_ imitating the original setting of two observable markers where this test was claimed to follow a ${\chi }_{1}^{2}$ distribution^14^.

**Figure 1:**
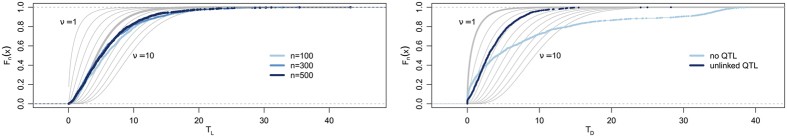
panel a: Empirical cdf of the *T*_*L*_ for data simulated under ${H}_{0}^{1}$. panel b: The empirical cdf of the *T*_*D*_ for data simulated under ${H}_{0}^{2}$ (navy curve), and data simulated under ${H}_{0}^{1}$ (light blue curve).

The empirical cumulative density function for *T*_*L*_ obtained from the simulation are shown in Fig. 1 (panel a) overlaid on several ${\chi }_{\nu }^{2}$ cumulative density functions for $\nu =\mathrm{1,}\ldots ,\,10$. Our study produces similar results to those of Knott and Haley^1^, but with more pronounced evidence that the distribution of the *T*_*L*_ statistic corresponding to the data simulated under the null hypothesis ${H}_{0}^{1}$ differs from the theoretical asymptotic result (${\chi }_{5}^{2}$) of Wilks^13^. For example, the mean, variance, and 95th quantile of the 1,000 *T*_*L*_ statistic obtained from the simulation were respectively 6.9, 28.6, and 17.8. Those summaries of the ${\chi }_{5}^{2}$ distribution are roughly 5, 10, and 11.1, respectively. Therefore, far more than 5% of the *T*_*L*_ statistic would be in the critical region (roughly 16.5%) when using the ${\chi }_{5}^{2}$ distribution to obtain the threshold. In other words, the type I error rate would not be well controlled.

As for the test of ${H}_{0}^{D}$ (navy curve of the panel b of Fig. 1), our simulations confirm that the ${\chi }^{2}$ assumption of current literature appears to be correct only in the case that both the marker and QTL genotypes are observable when the data is generated under ${H}_{0}^{2}$. However, the distribution of *T*_*D*_ is extremely poorly behaved (light blue curve of the panel b of Fig. 1) when the data is generated under ${H}_{0}^{1}$. This poorly behaved distribution emphasizes why inventing a new hypothesis testing approach is necessary, and also restates the open statistical questions that we discussed in previous subsections. Note that the QTL is not observed in real data analysis, so ${H}_{0}^{2}$ cannot represent the true situation of the null in any real data analysis, but only ${H}_{0}^{1}$ can.

In summary, two marginal distributions of the test statistics *T*_*L*_ and *T*_*D*_ in the LD-based QTL model both fail to follow the previously suggested asymptotic ${\chi }^{2}$ distributions. Additionally, the challenges will be even larger if performing a bivariate test statistic (*T*_*L*_ *, T*_*D*_ *)* instead of two individual marginal tests because the situation in two dimensional space is generally harder than that of one dimensional space; and the joint distribution of the bivariate test statistic (with strong correlation structure) is completely unknown.

## Methods

In this section, we describe our proposed approach. It considers the joint distribution of the two aforementioned test statistics (*T*_*L*_,*T*_*D*_ *)* in the bivariate sense, using a simulation-based bivariate null kernel to determine appropriate joint significance thresholds for the bivariate test structure in two dimensional space, which we call the BNK (bivariate null kernel) approach.

### BNK scheme for one marker

Let (*T*, *U*) denote the bivariate test statistics of interest, whose components are not necessarily independent, for the bivariate null hypothesis $({H}_{0}^{T},{H}_{0}^{U})$. (The bivariate null hypothesis for the LD-based QTL model is $({H}_{0}^{L},{H}_{0}^{D})$ with bivariate test statistics (*T*_*L*_, *T*_*D*_)). Here, we use a general version of notations to enable the BNK approach to easily extend to general contexts without being restricted by the QTL field. The approach is performed with the following steps.

- Step 1: Randomly simulate *k* null data sets, each of sample size *n*, following the model structure under bivariate null $({H}_{0}^{T},{H}_{0}^{U})$.
  The corresponding bivariate null model for the LD-based QTL context is likelihood (3), and phenotype ${Y}^{\ast }$ is simulated from the normal distribution with sample mean $\bar{Y}$ and sample variance ${\hat{\sigma }}^{2}$, as estimated from the observed phenotype data. Marker ${M}^{\ast }$ is simulated from the multinomial distribution with allele frequency $\hat{p}$, as estimated from the observed marker data. Marker and phenotype should be simulated independently.
- Step 2: Calculate test statistics (*t*_*i*_, *u*_*i*_) for each simulated null dataset, *i* = 1, …, *k*.
  For the LD - based QTL context, the maximum likelihood estimates of all unknown parameters are produced from the EM algorithm^11,12^, and then the bivariate test statistics $({t}_{Li},{t}_{Di}),\,i=1\ldots ,k$ are computed accordingly from equations (4) and (5), using simulated marker-phenotype data $({Y}_{i}^{\ast },{M}_{i}^{\ast })$.
- Step 3: The resulting *k* pairs of test statistics (*t*_*i*_, *u*_*i*_) computed from the second step will be used as the basis for estimating a bivariate null kernel distribution. That is, estimate the bivariate joint density function, $\hat{f}(T,U)$, using a null kernel density estimation technique based on (*t*_*i*_, *u*_*i*_), *i* = 1, …, *k* (see, for example, the bivariate.density function of the sparr package^24^ in R). The bandwidth should be selected so that the size of the bivariate test is maximized while still being less than or equal to $\alpha =0.05$ (or any desired significance threshold). In simulation studies, we will demonstrate that the type I error rate is controlled well when choosing the bandwidth in this way.
- Step 4: Compute the cdf $\hat{F}$ of $\hat{f}$ by $\hat{F}(c)={\int }_{A(c)}\hat{f}$, where $A(c)=\{(t,u)|\hat{f}(t,u)\ge c\}$.
- Step 5: The bivariate test $({H}_{0}^{T},{H}_{0}^{U})$ is rejected only if the value of the computed test statistics $({t}^{\ast },{u}^{\ast })$ for observed data falls beyond the acceptance region in the two-dimensional space. Let ${C}_{\alpha }$ be the non-negative value such that $1-\alpha =\int {\int }_{A({C}_{\alpha })}\hat{f}(t,u)dtdu$. Then the acceptance and rejection regions are separated by the ${C}_{\alpha }$ level contour of the estimated joint density function $\hat{f}$, under significance level $\alpha$. The joint *P*-value for $({t}^{\ast },{u}^{\ast })$ can then be obtained by the formula $p=1-\hat{F}(\hat{f}({t}^{\ast },{u}^{\ast }))$. The *P*-value obtained in this manner thus represents the probability under $({H}_{0}^{T},{H}_{0}^{U})$, as estimated by $\hat{f}$, that joint test statistic (*T,U*) would be more extreme than, or less likely to occur than, the observed joint test statistic $({t}^{\ast },{u}^{\ast })$. While two tests are involved, only one single *P*-value for each pair of tests is the output of this bivariate test structure, which enables it to connect with multiple correction approaches. For example, the P-values for all genetic markers can be passed to Holm’s procedure^25^ to strongly control the family-wise error rate.

When applying the BNK approach to the LD-based QTL model, only the normality assumption for phenotype is crucial; these are actually the default assumptions for any quantitative traits. The most crucial part of BNK approach described in Step 3 is a nonparametric approach without making any assumptions or requiring the asymptotic or theoretical distribution s of test statistics to be known. The number of simulated null data sets (i.e. *k* in the first step; also the basis used to fit the null kernel density in the third step) will not change results much, once it is large enough; larger *k* certainly provides a more accurate result but also carries a heavier computational burden. One should make a decision on *k* considering the balance of speed and resolution, exactly as is done when choosing how many permutations to perform in permutation testing.

### BNK scheme for genome-wide data

Besides the aforementioned challenges that exist for one marker, there are more challenges for the entire genome-wide data. The allele frequencies of markers in the whole genome can vary dramatically between zero and one. In order to overcome these challenges and make our approach extendable to whole genome data, we grouped the markers into several packets according to their allele frequencies and perform BNK for each packet separately.

Let *P* denote the total number of markers along the entire genome. The key steps of BNK for the entire genome-wide data are outlined as follows.

- Step 1: Group the *P* markers into 20 packets, according to their estimated allele frequencies, $\hat{p}$, as $\mathrm{(0,}\,\mathrm{0.05],}\,\mathrm{(0.05,}\,\mathrm{1],}\ldots ,$ $\mathrm{(0.95,}\,\,\mathrm{1]}$, which gives a meticulous division.
- Step 2: For each packet, simulate the null marker data ${M}^{\ast }$ using $\bar{\hat{p}}$, the mean of each packet’s estimated marker allele frequencies. Perform one BNK procedure (i.e. obtain one $\hat{f}$ and $\hat{F}$) for each packet, and then compute a *P*-value for each of the markers belonging to each packet. Repeating this step for all 20 packets will result in *P*-values for all markers along the entire genome.
- Step 3: Adjust the resulting marker *P*-values using Holm’s procedure^25^ to strongly control the family-wise error rate at $\alpha =0.05$.

While choosing more than 20 packets would theoretically improve precision, in actual practice we have found excellent agreement in results using this computationally convenient approach and results from the computationally-expensive per-marker BNK implementation.

## Results

To assess the performance of our newly proposed BNK approach, we work on one real genome wide association study (GWAS) dataset and three quite different simulation settings to give a comprehensive evaluation.

### Real GWAS data analysis

**Figure 2:**
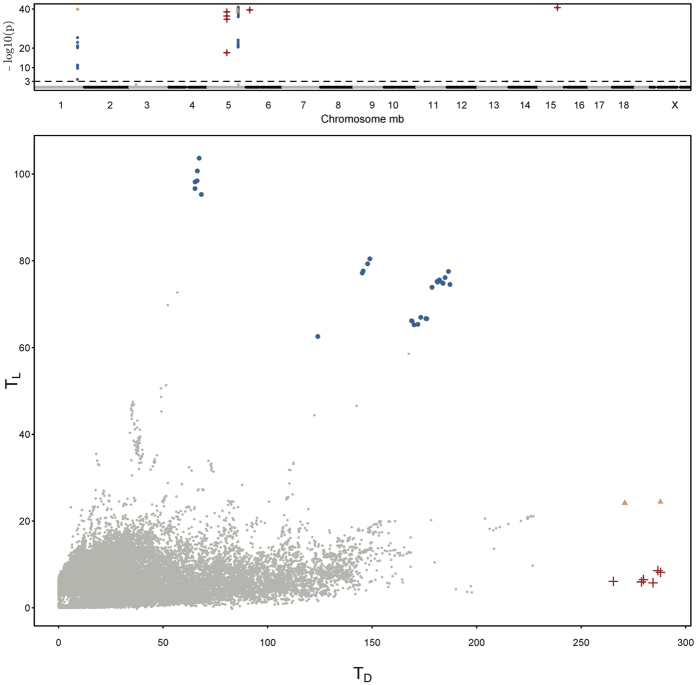
Top panel: the Manhattan plot with negative log of the Holm adjusted *P*-values for the BNK approach across all of the 44,428 markers from the mice HDL GWAS data. Bottom panel: The bivariate plot of the observed test statistics (*T*_*L*_, *T*_*D*_) for the same dataset. Blue points (linked with the marker and strong QTL effect), red crosses (strongly linked but weak QTL), and orange triangles (strongly linked but medium QTL) denote markers that were found significant under the BNK approach.

We applied BNK to a publicly available mouse high density lipoprotein (HDL) GWAS dataset^26^. The data (http://cbd.jax.org/datasets/datasets.shtml) contain 44,428 distinct markers spanning the entire mouse genome for *n* = 0.288 individual outbred mice. Measurements of HDL cholesterol were obtained for each mouse with the intention of mapping QTL responsible for HDL. The bottom panel of Fig. 2 shows the bivariate view of the two test statistics, (*T*_*L*_,*T*_*D*_), for each of the 44,428 markers analyzed. The top panel of Fig. 2 is the Manhattan plot with negative log of adjusted *P*-values across all markers and chromosomes. The most attractive property of this BNK approach is that the vast majority of markers are shown to have no direct effect on the trait, and their P-values fade away when they should, and only five influential peaks stand out dramatically (Fig. 2, top panel). The colors of the bottom and top panel are consistent.

The points in blue (‘linked with the marker and strong QTL effect’), orange triangle (‘strongly linked with the marker but medium QTL effect’) and red cross (‘strongly linked with the marker but weak QTL’) demonstrate those markers which were found significant after the Holm adjustment on the raw *P*-values obtained from the BNK approach. Four standard QTL approaches in current existing literature, the trend test, ANOVA test, linear mixed model, and the two univariate tests (not the bivariate version) assuming ${\chi }^{2}$ for QTL existence test and linkage test between marker and the QTL, have been implemented to analyze the same dataset^12,26^. They all reported exactly the same positions as those ‘linked with the marker and strong QTLs’ (in blue) points detected by BNK. These ‘linked with the marker and strong QTLs’ points correspond to two strong blue signals on Chromosome 1 at the 173 Mb position (172.9–173.2 Mb) with p-value 10^–12^ and on Chromosome 5 at the 125 Mb position (124.7–125.6 Mb) with p-value 0. The QTL at Mb125 of Chromosome 5 is named *Hdlq1*, and the causal gene underlying it is *Scarb1* (a well–known gene involved in HDL metabolism), confirmed by haplotype analysis, gene sequencing, expression studies, and a spontaneous mutation^26,27^. Additionally, numerous mouse crosses have linked HDL to Chr 1’s locus at Mb173, underlying which the *Apoa2* gene has been highlighted in Flint and Eskin’s research^26,28^.

Besides the unanimously ‘linked with the marker and strong QTL effect’ in blue, the BNK approach is also able to report a few extra findings beyond the limits of traditional approaches, as follows:

First, in traditional univariate QTL existence test, a *T*_*L*_ value around 20 yields a p-value close to $5\,\ast \,{10}^{-5}$, which fails to be rejected by any univariate test approach after multiple correction of 44,428 markers (i.e., $\mathrm{0.05/44428}=5\,\ast \,{10}^{-6}$). As a result, the orange triangles in Fig. 2 in the vicinity of the aforementioned peaks of Chr1 and Chr 5 have been missed by previous traditional approaches. However, their *P*-values obtained from the BNK approach are 0s and their test statistics clearly stand out in the two dimensional space. In particular, we found that the marker located at 173.3 Mb of Chr 1 lies within the *Aim2* gene (173.3–173.4 Mb) and the marker located at 122.1 Mb of Chr 5 lies within the *Ccdc63* gene (122.10–122.14 Mb). If only performing one hypothesis test (${H}_{0}^{L}$) as traditional *r*-based QTL models did (i.e. only looking at the vertical direction in the bottom panel of Fig. 2), the *P*-values of orange triangles would be larger than any of those blue points because their *T*_*L*_ values are smaller. However, considering the joint distribution of (*T*_*L*_,*T*_*D*_) for the bivariate test $({H}_{0}^{L},{H}_{0}^{D})$ from the two dimensional aspect, it is possible that the *P*-value of a marker with moderate QTL effect would be smaller than that of a marker with strong QTL effect if its joint effect is stronger. In the genetics literature, it is very important but harder to allow detection of multiple medium-effect linked QTLs^3^.

Second, we detect a few significant markers (in Fig. 2, red crosses), whose *T*_*L*_ values are extremely small but *T*_*D*_ values are extremely large. These red points correspond to three new (red) peaks that are not reported by existing literature for the same dataset yet are found to have a few genes reported to be important for the homeostasis/metabolism expression underlying them. Specifically, the markers located at 793.7–796.7 Mb of Chr 5 lies within the *Dshv2* gene (751.5–978.7 Mb); marker located at 207.6 Mb of Chr 6 lies within the *Dbts1* gene (182.3–733.8 Mb); and the marker located at 783.7 Mb of Chr 15 lies in the *Cocia2* gene (725.0–800.1 Mb) and *Scfq2* gene (346.0–787.1 Mb). We understand that researchers who have extensively used traditional QTL approaches may be initially reluctant to conclude significance when they see a *T*_*L*_ value close to 0, if they typically only perform one test and would be inclined to only consider the vertical direction in the bottom panel of Fig. 2. However, after close inspection, we noticed that these markers have extremely small minor allele frequencies (MAF; called “low-frequency variants” if 0.5%< MAF <5% and “rare variants” if MAF <0.5%). These low-frequency variants and rare variants have been emphasized as the frontier of future studies in the genetic basis of human diseases^29–31^. Therefore, we believe that the red points in Fig. 2 require further molecular genetics studies before any definitive interpretations can be made.

Note that the new genes are recognized from the Mouse Genome Informatics (MGI) webpage on February 11, 2017 (http://www.informatics.jax.org/marker).

### Simulation setting 1

In this subsection, we assess the type I error rate and power of BNK via simulation based on the LD based QTL model, from which the BNK is motivated. The type I error rate was evaluated via simulations generated under the null hypothesis of “no QTL”. Specifically, phenotypic data *Y* was simulated from a single normal distribution with a mean of 10 and unit standard deviation. In each simulation, a marker *M* was generated independent of *Y* with allele frequency 0.5. Additionally, we also performed a separate power assessment via simulations generated under the alternative hypothesis of “linked QTL”. The true QTL (*q* = 0.7) was generated under a conditional probability given the simulated marker *M* (*p* = 0.5) and linkage disequilibrium (*D* = 0.08) between marker and QTL. Then phenotype *Yi* was generated from a mixture normal distribution taking into account the information of the true QTL.

One hundred simulations were repeated in their entirety with varied sample sizes of *n* = 100, 300, and 500. *P*-values less than $\alpha =0.05$ were called significant. The results of these 100 simulations are included in Table 1. These results demonstrate that the BNK approach is indeed a conservative level $\alpha$ test, with type I error rate less than 0.05 under various sample sizes even when *n* = 100. The power of BNK is able to achieve 98% for an adequate sample size.

### Simulation setting 2

Since the traditional *r*-based QTL models are so widely used, we want to make sure that BNK also works well on exactly the same interval mapping of *F*2 design that was purposely designed for the traditional *r*-based one-test QTL model^4^. A simulation was performed using the sim.map and sim.cross functions of the R/qtl package^32^. Similar to Churchill and Doerge^4^, four chromosomes were simulated under a sample size of *n* = 100 individuals, with the first and third chromosomes having 50 markers each and the second and fourth having 10 markers each. All chromosomes were assigned a length of 100 cM. Two QTLs were simulated, one on the first chromosome at 44.4 cM (from the left end) and the other on the second chromosome at 61.6 cM (from the left end). The first QTL was given an additive effect of 0.75 ($\sigma =1$) and the second an additive effect of 1 ($\sigma =1$).

**Figure 3:**
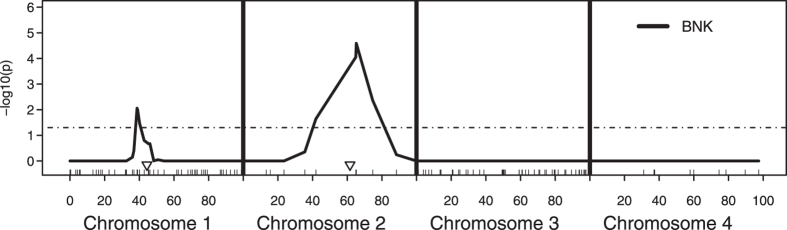
The resulting adjusted −log_10_ *P*-values obtained from the BNK approach for the simulated QTL interval mapping example. The dash line is the cutoff threshold for significance level 0.05. The rug plot shows the locations of the simulated markers, with triangles indicating true QTL.

The resulting *P*-values obtained from the BNK approach are adjusted using Holm’s procedure^25^ to control (strongly) the family-wise error rate. Figure 3 demonstrates the results of this simulation by plotting the negative log_10_ of the Holm-adjusted *P*-values obtained from the BNK approach across each marker and each chromosome. As is seen, BNK successfully find s the most significant QTL on chromosome 2. For the other QTL that was purposely designed to be weak on Chromosome 1, BNK just moderately passes the threshold as expected. The most important point is that BNK detects successfully the only two true QTLs and at the same time pushes all non-QTL regions to clear non-significance (flatline of 0 in Fig. 3). In short, the BNK *P*-values stand out when they should (to guarantee power), and fade away when they should (to control the type I error rate).

### Simulation setting 3

To demonstrate proof-of-concept of a general application of BNK, we compare the BNK approach with the well–established Hotelling’s *T* ^2^ statistic under a more general scenario outside of the genetics context. We consider a very general example of testing equality of bivariate mean vectors, $({H}_{0}^{T},{H}_{0}^{U}):({\mu }_{1},{\mu }_{2})=({\mu }_{01},{\mu }_{02})$, in two dimensional space. Hotelling’s *T* ^2^ statistic is the multivariate generalization of the univariate t test^33^, with strict bivariate normality assumptions on the data set. Hotelling’s *T* ^2^ statistic is calculated by

$${T}^{2}=n(\bar{{\bf{X}}}-{\mu }_{0})^{\prime} {S}^{-1}(\bar{{\bf{X}}}-{\mu }_{0})$$

where ${\bf{X}}=({X}_{1},{X}_{2})$ is the bivariate normally distributed random vector with mean vector $\bar{{\bf{X}}}=({\bar{X}}_{1},{\bar{X}}_{2})$, ${\mu }_{0}=({\mu }_{01},{\mu }_{02})$ represents the hypothesized mean, and *S* denotes the sample covariance matrix. The main contribution of Hotelling is in demonstrating that the distribution of *T* ^2^ follows $\mathrm{2(}n-\mathrm{1)/(}n-\mathrm{2)}{F}_{\mathrm{2,}n-2}$ under $({H}_{0}^{T},{H}_{0}^{U})$. This provides the corresponding *P*-value as $p=P({T}^{2}\mathrm{ > 2(}n-\mathrm{1)/(}n-\mathrm{2)}{F}_{\mathrm{2,}n-2})$ ^33^. For demonstration purposes, we computed two marginal t-test statistics $(T=\sqrt{n}({\bar{X}}_{1}-{\mu }_{01})/{S}_{11},U=\sqrt{n}({\bar{X}}_{2}-{\mu }_{02})/{S}_{22})$, and then applied the general BNK approach. Here *S*_11_ and *S*_22_ are diagonal components of matrix *S*.

**Figure 4:**
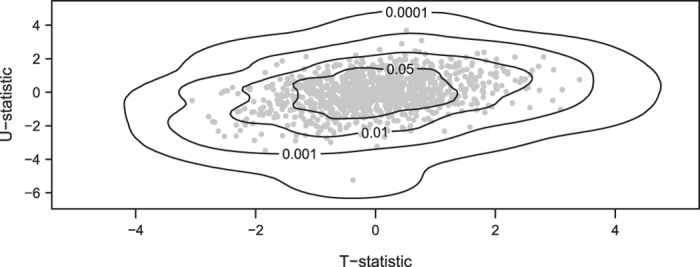
Visualization of the ${C}_{\alpha }$ level (or *P*-value) contours of $\hat{f}$ estimated by BNK from 1,000 (*T*, *U*) statistics for a bivariate normal simulation example with zero mean, unit variances, and covariance of 0.3.

To compare the Hotelling and BNK methods, each of the 100 replications of sample size n = 20 was simulated to perform the test $({H}_{0}^{T},{H}_{0}^{U}):({\mu }_{1},{\mu }_{2})=\mathrm{(0,0)}$. The data were generated from a bivariate normal distribution with mean $\mu =({u}_{1},{u}_{2})$ (${u}_{1},{u}_{2} \sim$ Unif(0,1) for a power simulation; in a separate type I error rate simulation, *u*_1_ = *u*_2_ = 0) and with covariance matrix $\sum =\mathrm{((1,0.3})^{\prime} ,\,\mathrm{(0.3,1})^{\prime} )$. An example of the estimated null kernel density $\hat{f}$ estimated by BNK for one replication, together with contours corresponding to various significance levels, are plotted in Fig. 4.

**Figure 5:**
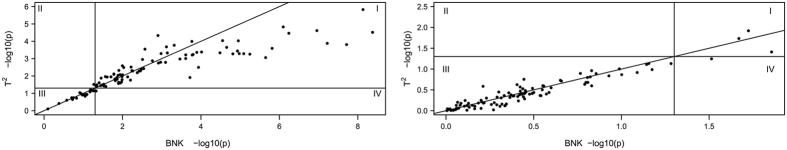
Power (panel a) and type I error rate (panel b) comparison of the −log_10_ of the *P*-values obtained from the BNK and Hotelling’s *T* ^2^ approaches for the general bivariate normal simulation that is outside of the genetics context.

Figure 5 shows how the 100 *P*-values from both the Hotelling and BNK approaches compare to one another. The horizontal and vertical lines are drawn at the critical threshold -log_10_(0.05). The panel a of Fig. 5 demonstrates the results of the power simulation. Quadrant I identifies tests declared significant by both approaches (power), and contains 79 of the 100 points. Quadrant III identifies those tests in which the null hypothesis is retained by both approaches (type II errors), and contains 19 of the 100 points. Thus, in 98 of the 100 simulated tests the two methods arrive at the same conclusions. Quadrant II and IV depict regions of discord, with one point located in each quadrant. The panel b of Fig. 5 depicts the results generated from the type I error rate simulation. The two approaches again agree for these simulations, with very similar trends shown in Quadrants I (the type I error area) and III (the correct area). Thus, both the BNK and Hotelling *T* ^2^ approach properly control the type I error rates, with less than 5% of the simulations resulting in type I errors. Specifically, the BNK approach reports a 4% type I error rate while the Hotelling *T* ^2^ approach reports a rate of 3%.

Although these two methods show good agreement in this bivariate normality simulation, the BNK approach still has some advantages over Hotelling’s *T* ^2^. The BNK approach not only works well for the Bivariate normal data that Hotelling’s *T* ^2^ is restricted to (e.g., simulation setting 3), but also works well for genetics context, where the phenotype data are normally distributed but marker data are categorical (e.g., simulation settings 1 and 2). For situations where the sampling distribution of test statistics cannot be obtained accurately, as in the case of the QTL model, BNK may work well but Hotelling’s *T* ^2^ would be not applicable.

## Discussion

As reviewed in Doerge^3^, early successes in QTL mapping in experimental populations ranged from the location of the cystic fibrosis gene in humans, to the identification of a gene affecting horn development in cattle, and further studies have continued to reveal findings as diverse as QTL impacting fruit texture in apples. Despite great success in the past, traditional QTL approaches have limitations that prevent them from being directly applied to new genotyping technologies. This manuscript presents an innovative data-driven statistical approach, BNK (bivariate null kernel), that overcomes several limitations of traditional QTL approaches.

- First, BNK provides a comprehensive understanding of the entire genome by closely and visually displaying the relationship of ‘linkage’ and ‘QTL effect’ in two-dimensional space. As demonstrated in bottom panel of Fig. 2, the genomic regions are roughly categorized as ‘weakly linked but strong QTL’, ‘strongly linked and strong QTL’, ‘strongly linked and medium QTL’, or ‘strongly linked but weak QTL’ to provide a comprehensive understanding of the entire genome. This information will guide researchers in molecular genetics, plant genetics, agriculture and other fields to greatly increase their experiments’ efficiency^7^.
- Second, the new findings discussed in the “Real GWAS data analysis” subsection demonstrate the great potential of the BNK approach. The low-frequency or rare variants, and multiple variants having moderate effects which have been beyond the detection ability of the majority of traditional QTL approaches, have been reported as functional contributors to complex disease risk, and are crucial for finding the missing heritability of complex diseases^29–31^.
- Third, the BNK approach works not only for experimental and domesticated species, but also for natural populations and rarely-studied new species. It works for the data collected not only from simple classic cross designs such as F2 intercrosses or backcrosses, but also collected from complex cross designs such as inbred or outbred lines.
  Besides contributing to the genetics literature, this manuscript also contributes to the statistics literature.
- First, this article proposes an important statistical bivariate hypothesis testing method, driven by an applied genetics problem. No available statistical approach in existing literature is directly relevant to the motivating two-test genetics problem. Permutation testing has become the gold standard in hypothesis testing in the QTL field whenever the distribution of test statistics is unknown or inaccurate^4^. However, the permutation testing approach was designed purely for a univariate test statistic structure (*T*_*L*_) and is not applicable to a two-test-statistics structure (*T*_*L*_, *T*_*D*_). The BNK approach is advantaged in the strict dichotomy between significance and non-significance in the resulting adjusted *P*-values. BNK *P*-values stand out when they should (to guarantee power), and fade away when they should (to control the type I error rate). As demonstrated in Fig. 3, the only loci for which any significance is found in the adjusted *P*-values relate very well to the true (simulated) QTLs. Every other loci was identically 0 in the adjusted -*log*_10_ *P*-value. This strict dichotomy property is crucial to guarantee the nice performance for a hypothesis testing approach.
- Second, this article resolves two open statistical issues – the unidentifiability of one parameter under testing, and the inaccurate theoretical sampling distributions when dealing with one hidden QTL and one observable marker. Both of these issues inflate the type I error rate and increase the difficulties of the hypothesis testing process. The BNK approach characterizes the joint distribution of the bivariate test statistics non-parametrically without requiring the marginal distributions of test statistics to be accurate or known, controls the type I error rate well below $\alpha$, and hence resolves these open issues.
- Third, the comparison of BNK and Hotelling’s *T* ^2^ approaches in the third simulation setting demonstrate the further general application ability of BNK. By testing equality of any general bivariate mean vectors when the data follow a bivariate normal distribution, the BNK approach has the potential to be applied to finance, biology, pharmaceutics, nutrition, and so on. Although it is motivated by a genetics problem, it shows potential to other fields outside of the genetics field. While the BNK approach could be compared to existing multivariate tests of location besides Hotelling’s *T* ^2^ test^34,35^, such a comparison is outside the scope of this manuscript. Instead, the comparison with Hotelling’s *T* ^2^ is presented in this manuscript only as a proof-of-concept (as stated at the beginning of the ‘Simulation setting 3’ section) to establish the general viability of the BNK approach, and we emphasize its application in the QTL context with bivariate hypothesis testing.
- Fourth, this article touches on quite a few interesting statistics topics such as bivariate hypothesis test ing, null kernel density estimation, mixture model, hidden variable, unidentifiability, and the correlation between one hidden variable and one observable variable.
